# Usefulness of a laparoscopic approach for treatment of small-bowel obstruction due to intersigmoid hernia: a case report

**DOI:** 10.1186/s40792-017-0298-y

**Published:** 2017-02-04

**Authors:** Norisuke Shibuya, Mitsuru Ishizuka, Yoshimi Iwasaki, Kazutoshi Takagi, Hitoshi Nagata, Taku Aoki, Keiichi Kubota

**Affiliations:** Second Department of Surgery, Dokkyo Medical University, 880 Kitakobayashi, Mibu, Shimotsuga, Tochigi 321-0293 Japan

**Keywords:** Intersigmoid hernia, Laparoscopic surgery, Long tube

## Abstract

It is well known that intersigmoid hernia (ISH) is a rare condition. Here we describe our experience of laparoscopic surgery for small-bowel obstruction (SBO) due to ISH after sufficient decompression involving long-tube insertion.

A 45-year-old woman with no history of abdominal surgery visited our hospital with epigastric pain. She was diagnosed as having SBO and underwent long-tube insertion as conservative therapy. However, her symptoms did not improve. Gastrografin contrast enema via the long-tube demonstrated a beak sign in the lower left abdomen and CT showed incarcerated small bowel was successively covered by sigmoid mesocolon, suggesting that the SBO was due to ISH, and she underwent laparoscopic surgery after sufficient decompression of the dilated small bowel.

Intraoperative examination demonstrated incarceration of a loop of the small bowel in the intersigmoid fossa without strangulation. Because the incarcerated portion of the small bowel was not necrotized, herniation repair was performed by removing the incarcerated small bowel from the intersigmoid fossa without closure of the hernia orifice.

The postoperative course was uneventful, and the patient is now free of symptoms and recurrence 12 months after surgery. Laparoscopic surgery after sufficient decompression is a useful treatment for SBO due to ISH.

## Background

Internal hernia is one of the causes (0.5–0.8%) of small-bowel obstruction (SBO) in patients with no history of abdominal surgery [1]. Among the internal hernias, intersigmoid hernia (ISH) is a rare condition and difficult to diagnose before surgery. Because patients with ISH have few specific clinical and radiographical signs, it is often diagnosed after laparotomy.

Here we describe a case of ISH that was diagnosed before surgery using gastrografin contrast enema via an inserted long tube and CT findings. The patient underwent laparoscopic surgery after ensuring sufficient decompression of the dilated small bowel and made an uneventful recovery.

## Case presentation

A 45-year-old woman with epigastric pain was admitted to our hospital. She had a history of Hashimoto’s disease and depression, but had never undergone abdominal surgery. Upon admission, her vital signs were normal. Although she had bowel distension with left lower abdominal tenderness, there was no evidence of peritonitis. Her laboratory data were almost within normal limits except for slightly increased white blood cell count (12.5×10^3^ /mm^3^).

**Fig. 1:**
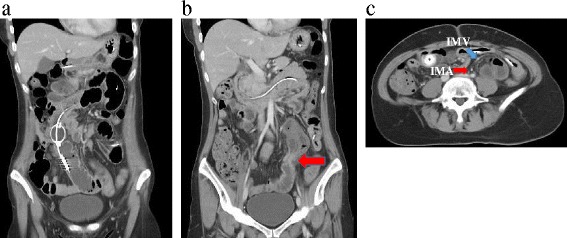
**a** CT shows dilation of the small bowel on the oral side of the obstruction. **b** Obstruction (*arrow*) with a closed loop of the small bowel. **c** The incarcerated small bowel was successively covered by sigmoid mesocolon

**Fig. 2:**
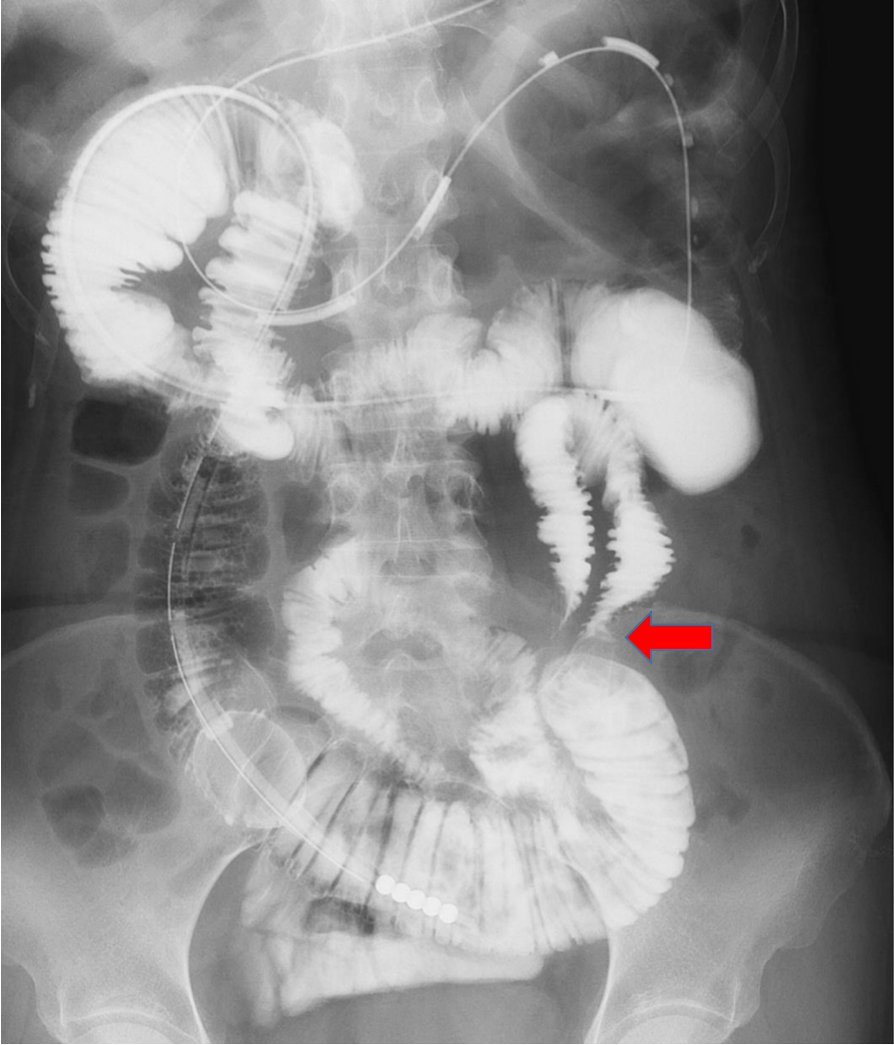
Gastrografin contrast enema through a long tube shows a closed loop of the small bowel with a beak sign in the mesosigmoid (*arrow*)

Because abdominal X-ray examination had demonstrated dilatation of the small bowel, we diagnosed the patient as having SBO and treated her conservatively by insertion of a long tube. However, this decompression therapy did not improve the symptoms. Computed tomography (CT) revealed obstruction of the small bowel in the left lower abdomen with small-bowel dilatation on the oral side (Fig.1a, b) and axial CT view findings clearly showed that the incarcerated small bowel was successively covered by sigmoid mesocolon. In fact, vessels of sigmoid colon run on the surface of sigmoid mesocolon (Fig.1c). Therefore, we diagnosed this herniation as ISH rather than internal hernia due to mesenteric defect. Gastrografin contrast enema examination demonstrated a closed loop of small bowel with a beak sign in the left lower abdomen (Fig. 2), suggesting that the SBO was due to an ISH. Therefore, after sufficient decompression, the patient underwent laparoscopic surgery 14 days after admission.

**Fig. 3:**
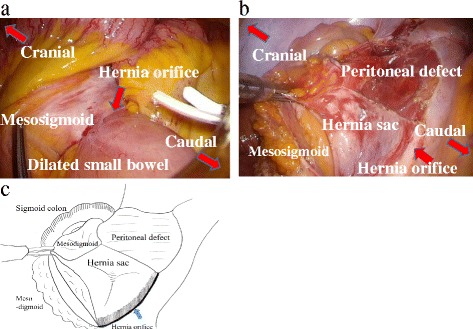
Intraoperative findings. **a** The small bowel was incarcerated in the intersigmoid fossa. There were mild adhesions between the incarcerated small bowel and the hernia orifice. **b** Adhesions of the mesosigmoid were removed from the hernia orifice. **c** An intelligible illustration

A 12-mm port for laparoscopy was inserted through the periumbilical region using the open technique, and an intra-abdominal pressure of 10 mmHg was established using carbon dioxide insufflation. Under laparoscopic observation, an additional 10-mm port was inserted through the right lower quadrant, and two 5-mm ports were also inserted through the right and left upper quadrants, respectively. Intraoperative observation revealed that the small bowel was incarcerated within the intersigmoid fossa, and there was no adhesion or fluid collection which suspected pelvic infection. Because the incarcerated portion of the small bowel was not necrotized, it was possible to remove the small bowel from the hernia orifice without resecting the former (Fig. 3a, b). After mobilization of the sigmoid colon, the orifice of the intersigmoid fossa was enlarged to prevent any recurrence of incarceration (Fig. 3c). The total operation time was 108 min, and the postoperative course was uneventful. The patient was discharged on the 8th postoperative day and is currently free of symptoms and recurrence of herniation at 12 months after surgery.

## Discussion

Internal hernias are defined as protrusion of a viscus through a normal or an abnormal peritoneal or mesenteric aperture within the confines of the peritoneal cavity [2]. Internal hernias account for about 0.5-5.8% of all cases of SBO. Herniation involving the sigmoid mesocolon accounts for about 6% of all internal hernias [2].

Benson and Killen classified herniation of the sigmoid mesocolon into three types: (1) intersigmoid hernias, (2) transmesosigmoid hernias, and (3) intramesosigmoid hernias [3]. On the basis of this classification, the present case was classified as an intersigmoid hernia (ISH).

The intersigmoid fossa is located in the area where the sigmoid colon turns from an upward direction to the right, where there is an attachment of the mesocolon to the posterior abdominal wall. Although it is a distinct and fairly constant structure, a recent study demonstrated it in 65% of autopsy cases [4]. However, individuals with this structure do not develop internal hernia routinely, because incarceration of the small bowel behind the mesosigmoid is necessary. Therefore, this type of internal hernia may have other causes such as elongation of the mesentery of the small bowel or incarceration of the small bowel within the pelvic space [5].

A recent report revealed that preoperative differentiation of these three types of hernia involving the sigmoid mesocolon is difficult, and the diagnosis is confirmed only with surgical management in most cases [6]. However, there is a typical formation in ISH that incarcerated small bowel loop and is formed from left lower abdomen to cranial side, because hernia orifice of ISH is located to the attachment of the lateral aspect of the sigmoid mesocolon [7]. Similarly, it is difficult to distinguish ISH from intramesosigmoid hernia (especially lateral type) using only CT findings, because incarcerated small bowel is formed from lower abdomen to cranial side in these two types of hernias [7]. Therefore, it is acceptable that final diagnosis should be defined by using intraoperative findings rather than preoperative CT findings in these two types of hernias.

On the other hand, it is important to distinguish transmesosigmoid hernia from the above two types of hernias because, transmesosigmoid hernia has not only a higher bowel resection rate (50%) than the other two types of hernias (ISH; 18.8%, intramesosigmoid hernia; 13.8%) but also a higher mortality rate (7.1%) (ISH; 0%, intramesosigmoid hernia; 0%) [8].

Therefore, laparoscopic surgery should be performed for patients with sigmoid mesocolon hernia, except for patients with transmesosigmoid hernia, because it is not difficult to distinguish transmesosigmoid hernia from other two types of hernias. In fact, patients with transmesosigmoid hernia require shorter time from symptom onset to operation (2.2 ± 3.2 days) than those with other two types of hernias (ISH; 7.7 ± 7.0 days, intramesosigmoid hernia; 11.8 ± 8.4 days) [8].

Thus, laparoscopic surgery is acceptable for patients with ISH, and intraoperative observation is useful to distinguish ISH from intramesosigmoid hernia.

Recently, laparoscopic surgery has been broadly performed for SBO. Laparoscopic surgery has not only high diagnostic value but also minimal invasiveness in comparison with open surgery. In particular, because ISH has few specific clinical findings or symptoms, preoperative diagnosis can be very difficult. Therefore, laparoscopic surgery is recommended for not only treatment but also diagnosis of SBO due to internal hernia, because it is not rare for internal hernia to be diagnosed after laparotomy.

Between 2002 and 2014, a total of 19 cases of ISH were reported from Japan. Among the patients, 7 (37%) were diagnosed before surgery and 3 (16%) underwent bowel resection because of obvious small-bowel necrosis. Therefore, most patients with ISH do not require small-bowel resection [9]. In addition, 15 patients (79%) underwent closure of the hernia orifice for prevention of hernia recurrence.

Therefore, with regard to the indication for small bowel resection, it should be performed when obvious small bowel necrosis was found or the color of small bowel was not so good. However, because intraoperative observation cannot evaluate the intraluminal change of small bowel, we correctly estimate the blood flow of small bowel by using a fluorescence imaging instrument [10] when we cannot define the indication of small bowel resection on the basis of intraoperative findings.

With regard to the indications for closure of the hernia orifice, we consider that if, on the basis of intraoperative observation, the hernia orifice appears small and there is a possibility that incarceration of the small bowel may recur, the hernia orifice should be closed. On the other hand, if the hernia orifice is large and there is no possibility of the small bowel becoming incarcerated, then it is acceptable not to close the hernia orifice. In the present case, we enlarged the hernia orifice to prevent recurrence of incarceration after mobilization of the sigmoid colon.

In addition, with regard to the reason of small bowel incarceration to the fossa of sigmoid mesocolon, it is supposed that estimating the reason of small bowel incarceration is difficult, because intraoperative observation revealed that there was no concrete evidence to distinguish congenital one from adhesion due to infection in the case.

During operation, we tried to retrieve incarcerated small bowel from intersigmoid fossa by tender operation, and we inserted the tissue pad of power device to the edge of hernia orifice to prevent small bowel injury, because incarcerated small bowel was fixed to the hernia orifice of sigmoid mesocolon.

Moreover, although long-tube insertion for treatment of SBO is not broadly recommended in Western countries, it may be needed in order to perform safe laparoscopic surgery with a sufficient space in the abdominal cavity, since it not only reduces the intraluminal pressure of the dilated small bowel but also facilitates correct preoperative diagnosis using gastrografin contrast enema. Similarly, even if resection of the small bowel is required, sufficient decompression using long-tube insertion is necessary to expedite not only safe anastomosis but also reliable reconstruction.

## Conclusion

Laparoscopic surgery after sufficient decompression of the small bowel is useful for SBO due to ISH.

## Abbreviations

CT: Computed tomography
ISH: Intersigmoid hernia
SBO: Small-bowel obstruction
